# Regulation of artificial intelligence in Uganda’s healthcare: exploring an appropriate regulatory approach and framework to deliver universal health coverage

**DOI:** 10.1186/s12939-025-02513-3

**Published:** 2025-05-30

**Authors:** Kalule Grancia Mugalula

**Affiliations:** Centre for Social Ethics and Policy (CSEP), Department of Law, School of Social Sciences, University of Manchester, Oxford Road, Manchester, M13 9PL UK

**Keywords:** Artificial intelligence, Regulation, Regulatory approaches, Universal health coverage, Uganda

## Abstract

**Background:**

Uganda, like other United Nations (UN) member states, has undertaken to achieve Universal Health Coverage (UHC) by 2030 in line with Sustainable Development Goal (SDG) 3 targets. However, if this target is to be achieved, efforts will need to be increased, as full coverage of essential services remains an issue. Access to quality, acceptable and affordable healthcare remains an illusion for many Ugandans. Artificial Intelligence can be a valuable tool in achieving UHC as it can increase access to health facilities in hard-to-reach areas. AI tools have also been reported to perform faster than humans at certain key health tasks like diagnosis. However, for AI to be effective in delivering its benefits, context-specific regulatory approaches are key, as needs and opportunities differ. In this paper, I argue that the regulation of AI can help make it an effective tool for achieving UHC in Uganda if the right regulatory approach and framework are adopted, as regulation shapes outcomes. This will tackle the risk of poor regulation hindering AI development and AI reinforcing inequalities.

**Methods:**

The paper employs a doctrinal methodology to analyse the two prominent regulatory approaches to AI in the EU and UK, which have adopted a risk-based and principles-based approach, respectively. It investigates whether these approaches are suitable for regulating AI in Uganda’s healthcare and achieving UHC. The strengths and weaknesses of each approach are examined. The paper advocates for considering a human rights-based approach that can be integrated with the principles-based approach.

**Results:**

Regulation is a double-edged tool that can positively or negatively shape health outcomes. Good regulation has the potential to emancipate ordinary people’s lives. Therefore, Uganda should leverage the positive aspects of principles-based and human rights-based approaches to regulation to ensure that AI’s potential to achieve UHC is effective.

**Conclusion:**

The hybrid approach to AI regulation is best suited to serve Uganda’s healthcare needs. The foremost priority for Uganda is to attain Universal Health Coverage. A hybrid approach will contribute to this however, it is not the silver bullet. Uganda needs to supplement efforts to achieve UHC with other non-regulatory strategies.

## Background

Good health is a precondition for sustainable development and an indicator of progress [1]. In recognition of this, the government of Uganda has, over the years, adopted several laws and policies both nationally and internationally, such as the Public Health Act, the National Health Policy I and II, and the Health Sector Development Plan, among others. These aim to improve access to healthcare and fulfil its obligations as a provider and financer of public services. This has seen an improvement in the population’s health as evidenced by reduced infant mortality rates, an increase in the number of children vaccinated by their fifth birthday and a reduction in maternal mortality rates [2]. Although this is laudable progress on some of the health indicators as seen in the Sustainable Development Goal (SDG) progress report [3], a lot still needs to be done to achieve Universal Health Coverage. This progress is mostly reported in urban areas and the health status of the rural population is still lacking in many respects. Suffice to note that, according to the World Bank, 73.84% of Uganda’s population lived in rural areas in 2022 [4].

Universal Health Coverage (UHC) aims to create a situation in which every individual can access the comprehensive array of necessary health services at the time and place they require, without experiencing financial difficulties [5]. The fundamental principles of UHC encompass the delivery of comprehensive, high-quality healthcare, ensuring equity and financial protection for all individuals, including those who are less advantaged. UHC transcends the mere provision of health services; it mandates that these services must be both accessible and of satisfactory quality for the entire population [5]. However, in Uganda, many public health facilities (which the rural populace predominantly relies on) have reported cases of absent medical staff, lack of emergency obstetric facilities, bribery and lack of essential medicines [6]. This highlights the poor quality of the services offered there [3]. In addition, although the private sector participation in health service delivery is not a new phenomenon, the continuous privatization and commercialisation of health facilities exacerbate the profit-driven motive behind the private provision of public services. The bulk of the well-stocked private health facilities concentrate their operations in the urban centres, making it a major concern, especially for availability and access to quality services for vulnerable groups and low-income households. This has left the right to health elusive for many Ugandans who do not receive proper treatment from public health facilities and cannot afford the services in private health facilities [7].

The fact that the existing public health facilities are largely underfunded and understocked means that they need more facilities to enable the poor and underprivileged to conveniently use them. The less privileged therefore, face difficulties in accessing quality and affordable health services [7]. Healthcare is expensive and inaccessible for the poor and vulnerable in rural areas, should they opt to seek treatment in private health facilities. The cost of an inpatient stay is approximately 75,000 Uganda shillings (equivalent to 15 pounds) per night, with extra charges applicable for any procedures or medications administered. This expense is beyond the financial reach of many individuals in Uganda, particularly given that the average monthly income is roughly 300,000 Uganda Shillings, which is equivalent to 60 pounds [8]. Medicines and interventions only have value if they reach the people who need them and do so in a way that ensures they are properly used. If the people who need healthcare cannot access it or afford it, UHC cannot be achieved. Healthcare incorporates many socioeconomic factors, as evidenced in the underlying determinants of health. The World Health Organisation notes that health is influenced by many factors such as genetics, behaviour, environmental and physical influences, medical care and social factors [9]. These affect an individual’s physical, mental, and social well-being. As the WHO Constitution states in its preamble, health goes beyond the absence of disease. The UN Committee on Economic, Social and Cultural Rights (CESCR) has outlined essential attributes to guide the implementation of the right to health, finding that health facilities, goods and services should be; available in adequate numbers, accessible –physically, economically without discrimination, in addition to being acceptable to all, respectful of medical ethics; and of good quality [10]. Thus, one of the most significant determinants of health is financial accessibility, yet poverty greatly hinders this. The SDG progress report indicated that Uganda was performing poorly on SDG 1 on ending poverty in all forms, and SDG 10 on reducing inequalities [3]. This is something that needs to be addressed if UHC is to be achieved.

Additionally, good health is also fundamental to the realisation of other rights, as expounded in the health capabilities approach, which highlights that one needs at least a basic level of health for a human life to flourish [11]. This approach stresses that health is important to live a flourishing life as without good health, one may not be able to realise other rights such as the right to work or the right to education. The capabilities approach also emphasises the significance of tackling the social and economic determinants of health, including poverty, education, and housing. It prioritizes individuals’ capacity to attain the aspects of life they deem valuable — highlighting the significance of human capabilities, including health, education, and social engagement, rather than merely concentrating on economic resources. Martha Nussbaum recognized several essential human functional capabilities that are fundamental for all citizens [12]. She enumerates Life, Bodily Health, and Integrity. The health capabilities approach also goes past biological rationalizations and demonstrates how social and economic factors impact health. It extends from solely evaluating health outcomes like mortality rates or disease prevalence and places focus on the capabilities that an individual has to attain good health and well-being [13]. This serves as an essential instrument for comprehending and tackling health disparities. By emphasising individuals’ capacity to lead healthy lives, it can contribute to the establishment of more equitable and just communities. Addressing inequalities in access to resources and capabilities that contribute to health disparities is, therefore, necessary. Thus, corresponding approaches, like reducing inequalities in access to health, are important to improve health and ensure that existing vulnerabilities are not exacerbated [14].

SDG target 10.3 provides that inequalities can be reduced by ensuring equal opportunities for all [15]. In healthcare, this necessitates the creation of conditions that guarantee access to medical services and care in instances of illness, and ensuring equal and prompt availability of fundamental preventive, curative, and rehabilitative health services, as well as health education [10].This also calls for eliminating discriminatory practices, laws, and policies and promoting appropriate ones. Uganda should, therefore, not relax based on the progress in urban areas [3] when the rural populace and SDGs that are key to achieving health are yet to be prioritised.

Consequently, as the race to achieve Universal Health Coverage by 2030 continues, Uganda needs all the strategies available to ensure she meets her commitment to this Sustainable Development Goal (SDG) target. Artificial Intelligence (AI) can be a helpful tool that improves access to health, especially in developing countries, where efforts to achieve UHC still need to be increased. AI can be a low-cost solution to filling gaps in expertise as compared to the number of trained clinicians needed to fill the workforce shortages. It possesses the capability to alleviate the human resources challenges in the healthcare sector by enhancing diagnostics, supporting decision-making processes, enabling big data analytics, and streamlining administrative tasks, among other functions [16]. The WHO reaffirmed the beneficial role that AI can play in realising UHC at the 2023 Global Conference on Digital Health tagged, ‘Taking UHC to the last citizen,’ It emphasized the need to harness digital technologies to accelerate progress towards achieving UHC [17].

Digital technologies like AI are shaping the future of healthcare. Artificial intelligence has been reported to perform better than humans at key healthcare tasks like analysis of health data and diagnosis [18]. It can also solve key health issues that have plagued the health system and troubled practitioners for years. In Uganda, for example, an AI-powered tracking device has been deployed by doctors in Mbarara Hospital (Western Uganda) to monitor mothers who have recently undergone caesarean births in a bid to reduce post-birth complications that have been resulting in maternal mortality [19]. Relatedly, the Infectious Disease Institute is using generative AI to aid the development of an interactional chat-based platform that will translate and communicate the national guidelines for pandemic preparedness into the different native languages [20]. It is hoped that this will aid non-English-speaking health workers in improving the management of pandemics. This is a limited reflection on how AI can be used to improve the diagnosis and treatment of diseases and to upgrade the efficiency of healthcare systems, more examples are given later in the paper.

The emergence of AI, however, poses both prospects and challenges. While it has the potential to address pressing issues like healthcare access, diagnosis and treatment of diseases, concerns exist about potential biases embedded in AI algorithms, data privacy violations, and the potential for AI to further marginalise vulnerable populations and exacerbate existing health inequalities [21]. In light of these harms, the question is not whether AI should be regulated but how AI should be regulated. It has been established that an effective regulatory framework is an essential tool in checking AI harms [22, 23]. A key question remains: What regulatory approach should be taken? The regulation of AI has attracted significant scholarly attention, with existing literature mainly focusing on the regulatory approaches taken by the European Union (EU), the United Kingdom (UK), and the United States of America [24–26].

However, little intellectual effort has been devoted to assessing the regulatory mechanisms that can lead to realising the full benefits of AI in developing and resource-limited settings. The Global Systems for Mobile Communications Associations (GSMA) regulatory impact assessment for Africa noted that emerging technologies are developing devoid of any explicit formal law designed wholly for their practice [27]. This necessitates not only a review of the existing regulatory frameworks but also an assessment of their adequacy in governing technologies like AI. Miller et al. highlight that e-pharmacy uses AI algorithms to streamline various processes involved in pharmaceutical operations like tracking inventory and management of prescriptions. Although the practice of online pharmacies increased greatly during the COVID-19 pandemic, it continues to grow without an established regulatory framework. They report that none of the countries in their study had enacted a regulatory framework specific to e-pharmacy [28]. This paper seeks to move beyond just e-pharmacy to other AI tools that can promote UHC. The regulatory space of many developing countries is often filled with legislative interventions based on global templates which sometimes ignore the recipient jurisdiction’s unique needs [29]. Such an approach is deficient in two respects. First, it does not recognise that needs and opportunities differ from country to country. Second, it does not recognise the importance of context-specific regulation in ensuring that AI benefits people in developing countries. Against this background, this paper seeks to contribute to filling this gap in the literature by answering the following question;

What is the most appropriate regulatory approach for enabling AI to deliver Universal Health Coverage in Uganda?

The scope of the paper is limited in two ways: First, it does not deal with an analysis of all AI regulation and regulatory approaches; it focuses its analysis on the two most prominent approaches to regulating AI, the risk-based approach to regulation as adopted by the EU in the Artificial Intelligence Act (‘AI Act’) and the principles-based approach as taken by the UK in the White Paper on AI regulation. The paper then makes a case for a hybrid of the principles-based and human rights-based approach. Second, the paper deals with AI in the healthcare sector only, specifically focusing on which regulatory approach can help leverage the benefits of AI to achieve UHC in Uganda.

In healthcare, regulation is a double-edged tool that can positively or negatively shape health outcomes. Regulation can provide benefits in terms of maintaining patient safety and quality of care. However, overlapping regulatory mandates can also cause inefficiency and compromise quality. Regulation can also be complicit in creating and contributing to health inequalities while simultaneously having the potential to address them. Oppressive laws and policies that result in health injustices can lead to a gradual decline in physical, mental, and emotional well–being. Yet well-shaped laws and policies that prohibit non-discriminatory practices and promote equality in access to services can promote health [29].

With efforts to achieve Universal Health Coverage still lagging in Uganda, this paper seeks to make a case for regulating AI in healthcare using a hybrid approach as one of the strategies to achieve UHC. The paper argues that regulation should be one of the vital players if AI is to contribute significantly to achieving UHC. In this it seeks to demonstrate the continuous influence of the law in shaping health, highlighting that the law can promote health and rights on the one hand and cause significant harm on the other [30]. The paper seeks to make a case for regulation of AI in healthcare on two grounds, first, that regulation can play a central role in ensuring equitable access and availability to the technology that will ultimately improve healthcare; and secondly, regulation can protect the public from the potential tech-related harms like bias and discrimination that the deployment of this technology poses. If AI is well-regulated, it has the potential to improve healthcare in Uganda but there is also a need to prioritise the safety of users. This can be achieved through a hybrid approach. As recognised by Braithwaite et al. good regulation has the potential to emancipate ordinary people’s lives [31]. Therefore, Uganda should leverage the positive aspects of regulation to ensure that AI’s potential to achieve UHC is effective.

For this to happen, the paper discusses the need for the right regulatory approach to be taken if AI is to help achieve UHC. With current regulatory approaches for AI in the EU and UK set between risk-based and principles-based approaches respectively, the paper analyses both approaches, highlighting the strengths and weaknesses of each approach. It then makes a case for a hybrid of principles-based and human rights-based approach that considers the Ugandan context. It supports the need for a hybrid approach, arguing that first, this will ensure that the country gets the best from regulation. Second, there is a need to recognise that every jurisdiction has distinctive social, economic, and cultural elements that may require customized regulatory approaches. It is therefore essential to choose a regulatory approach according to context as following a model based on a global template may not work to bring about the intended social and economic changes needed, which in the case of Uganda’s healthcare, is the achievement of UHC.

The paper consists of four sections. I begin with introducing and contextualizing UHC by discussing its components and how they stand in Uganda’s healthcare system. In the second section, I introduce the relationship between AI and UHC by showing how AI can benefit UHC and address some of the challenges to its achievement. The next section begins with an overview of the regulation of AI. I discuss the risk-based and principles-based regulatory approaches for AI adopted by the EU and UK respectively, highlighting some of the most pressing challenges in each regulatory approach. I then make a case for the human rights-based approach to regulation, arguing for Uganda to adopt a hybrid approach to regulating AI to achieve UHC. My concluding thoughts highlight a proposed framework to be taken.

To make a case for a hybrid regulatory approach as the gateway to achieving UHC in Uganda, it is essential to first understand the concept of UHC, its components, and Uganda’s status regarding each component. I thus proceed with this discussion as I highlight the challenges Uganda is facing in achieving each component.

### Understanding Universal Health Coverage

Universal Health Coverage envisions a situation where ‘all people have access to the full range of health services they need, when and where they need them without facing financial hardship’ [5] From the above definition, the core aspects of UHC are providing comprehensive quality care, equity and financial protection for all people, even the less privileged. It extends beyond just providing health services. These services should be of an acceptable quality and affordable for the entire population [32]. UHC is the pivot of Sustainable Development Goal (SDG) 3 as evidenced in Target 3.8 which highlights that there should be *access for all* to essential healthcare services, vaccines, and medicines that are safe, affordable, effective, and of good quality [33].

Although often viewed as a mere idealistic concept, that is ambiguous and lacks clarity [34], UHC has always been the overarching health goal as evidenced in the work of the WHO. At the Millennium Development Goals (MDGs) launch in 2000, the WHO highlighted that UHC was a global strategy for Health for all by 2000.WHO reiterated that this was not a return to the ideals of the declaration of Alma Ata but has continued to state that it is a cross-cutting strategy for realizing the Sustainable Development Goals (SDGs) and a practical expression of the right to health in International human rights law. Indeed, despite the criticisms of the MDGS, as having been narrow, selective, unequal, and lenient on states [35], globally health-related targets were realized over the years as evidenced in the halving of the global proportion of those living in extreme poverty and having no access to clean water [36].

Currently, the realization of SDG 3 is hinged on achieving UHC. The key components of UHC as highlighted above are access, equity, quality and financial protection. Through these four components, I give a glimpse of the problems in Uganda’s healthcare system, show how AI could address these problems and the challenges that would be faced.

#### Access

Access connotes both physical and economic access. Health services are said to be physically accessible when they are within reasonable reach for the people. This accessibility has to be universal in that everyone who needs healthcare can get it [5].Thus instances where those living in rural areas are ignored or have to walk long distances to reach the nearest health service are not permitted. They are financially accessible if people can access them without facing financial hardships [10].

In Uganda, both physical and financial access to healthcare remains a challenge. The health system consists of public and private sectors totalling 6,940 health facilities. The government owns only 45% of these, and 55% are owned by the private sector of which 40% are private for-profit and only 15% are private not-for-profit [37]. Public health facilities remain largely understocked, underfunded, and lack skilled staff. Those present have negative work attitudes due to low pay. The unmotivated and low numbers in the health workforce make it impossible to deliver and expand population-based health services which greatly hinders access [38]. It has been reported that in some villages, people walk up to 10 km to access the nearest health facility. Uganda has a National Minimum Healthcare Package that provides for access to free health services in all state-owned health facilities. It includes provision of sexual and reproductive health and rights, integrated management of childhood illnesses, control of communicable diseases and other public health interventions. It is well elaborated in the National Health Policies I and II and the National Development Plans (I, II and III) [39]. Unfortunately, the challenges in public health facilities impede the implementation of the health package. Additionally, the Ministry of Health developed a Policy establishing a program for community health extension workers in line with National Health Policy II [39]. These workers play a significant role in increasing access to integrated health services which are crucial in advancing public health service delivery [39]. However, to date, no deliberate efforts have been made to implement this program.

As noted earlier, the majority of private health facilities that are well stocked and function well have been established in the urban centres where viable business opportunities exist. This leaves the rural population at the mercy of the understocked public health facilities [6]. This fragmentation between the public and private sectors instead widens the healthcare access gap. Access to health by those living in rural areas has remained a challenge because of urban bias and transport-related challenges, leaving ‘less health centre access for the poorest residents with the highest health care needs [6]. Almost 72% of Ugandans experience prohibitive travel distances to health centres as they are not within one-hour walking distance of the nearest health centre II. Health Centre II is the first point of contact for medical treatment in government health facilities and provides outpatient care for common diseases in a parish or local community. In discussing the “growing outcome gap” between the populations receiving health interventions and those that do not, Farmer argues that, with the high costs of healthcare, it becomes problematic to receive equal healthcare as poor people will not receive the same treatment as the more financially fortunate [40]. Addressing these healthcare gaps is critical to attaining the SDG target for UHC.

#### Equity

Equity means *everyone* has access to the healthcare they need. Equity is an important component of Universal Health Coverage, especially for the less privileged. In line with the central focus of the SDGs to leave no one behind, this entails starting with the furthest behind, who are the marginalized, vulnerable and underprivileged. Thus, strategies to achieve UHC should be targeted at catering to their needs first. The health system in Uganda is criticised for being inequitable because the rich receive more healthcare than they need while the poor receive less healthcare than they need [41]. Inequalities in health are ethically concerning [42]. They have also been viewed as a public health concern and a human rights violation [42]. The distribution of the COVID-19 Vaccine highlighted this dilemma. In Uganda, for example, the government announced a deal for millions of vaccines from AstraZeneca, at $7 a dose – more than three times what the European Union (EU) paid for the same jab. With transport costs included the vaccine ended up costing $17 to fully vaccinate one Ugandan [43].This high expense culminated in the unequal distribution of vaccines and some people in rural areas were never vaccinated [44].

Additionally, equity in access to health is a major problem for women and children in rural settings, who have historically been prone to gender-related discrimination. They continue to face marginalization, various health inequities and many unmet health needs. This is worsened by the fewer economic opportunities and high rates of poverty [45]. The inequity in health can be highlighted in some of the cases that have been litigated. In the case of CEHURD vs. Attorney General and Nakaseke District Local Government Civil Suit No. 111 of 2012 a pregnant woman lost her life due to failure to access emergency obstetric care as the doctor was absent from the health facility. She was in obstructed labour for 8 h before dying. Similarly, in the case of CEHURD and Fatumah Nakayima vs. Director of Mulago National Referral Hospital and the Attorney General Miscellaneous Cause No. 327 of 2016, the hospital failed to provide information to a mother about the whereabouts of her child, the mother had given birth to twins but was only handed one baby. The hospital could not provide information on whether the second child was dead or alive, causing the mother mental and psychological trauma. The court found the government in violation of its obligations to respect, protect and fulfil the right to health in both cases. These cases reflect the inequity in the provision of healthcare services in public health facilities.

#### Quality

Quality means that healthcare services and facilities are timely, safe, effective, efficient, and people-centred. As noted above majority of the public health facilities especially in rural areas face staffing and financial challenges which affect the quality of healthcare provided there. While the WHO recommends that at least 4.45 health professionals are needed per 1,000 population to ensure that a country achieves UHC in line with the SDGs, currently only four African countries have achieved this [46]. Uganda stands at 1.87 per 1,000 population which is lower than the WHO standard [47, 48]. In addition, Uganda’s ever-growing population continues to strain the already low numbers in the health workforce. A WHO African Health survey indicates that the shortage of health workers is one of the main problems stifling the achievement of UHC in Africa [46]. The gaps in the healthcare workforce will require millions of dollars to be filled if Uganda is to achieve UHC by 2030.

A major funding gap exists in Uganda’s health sector which creates stumbling blocks for the achievement of UHC. Inadequate funding of health remains a global problem, especially for developing countries which in turn hinders the availability and accessibility of quality healthcare and increases out-of-pocket spending on health. Although African Union Countries agreed to spend at least 15% of their national budgets on health in the Abuja Declaration, health financing remains at a low scale in Uganda with the current budget being at only 7.7% [49]. There is also a lack of transparency regarding how funds are generated and distributed. A 2024 World Bank Press release highlights Uganda spends much less on health in comparison to her peers in the region. It thus notes that,

“Unless policymakers work to prioritize the health sector, Uganda is unlikely to achieve the health-related Sustainable Development Goals” [50].

Earlier, the World Bank had estimated that over half of the Ugandan population lives on less than a dollar a day [51].This means that the majority of Ugandans can neither afford health insurance nor health services in private health centres which are of better quality than public health facilities. According to WHO an increase in health spending simultaneously increases the UHC coverage index as it increases access and quality in healthcare [52].

#### Financial protection

Financial protection means being able to access healthcare without undergoing a financial burden, in that the funds spent on accessing health services do not threaten a person’s living standard or expose a person to economic hardship.

The health infrastructure in Uganda is still battling with the high cost of care. Out-of-pocket spending on health continues to be high for people who seek treatment in private health facilities [53]. Yet it is these facilities that have adequate and needed health services and facilities. Additionally, the lack of a national health scheme means out-of-pocket spending on health persists at very high rates [54]. It has been five years since the National Health Insurance Scheme Bill, 2019 was tabled in Parliament. The Parliament passed it into law in 2021 but the President has yet to assent to the bill so it can become law. Although as a matter of policy free healthcare services are available at government-funded health facilities, chronic underfunding of these facilities renders the services ineffective [55]. This means that people seek health care from private facilities that require out-of-pocket payments. The lack of a national pre-payment system means that Ugandans are not protected from the financial risk resulting from out-of-pocket healthcare payments [55]. Only a small majority of Ugandans (those whose employers provide the package as part of the employment terms) have enrolled for voluntary health insurance [56]. Undoubtedly, the financial risk associated with healthcare demands will only increase as the population increases. The situation is worsened by the fact that only employers in urban centres provide these benefits. People living in rural areas are left at the mercy of the understocked, underfunded public health facilities. An equitable and effective national insurance program is therefore urgently needed. It should be noted that a comprehensive national health law is key to ensuring that everyone in the country is eligible for the full package of health services, medicines and vaccines. No one should be excluded irrespective of income, gender, race, legal residence, or other status [30]. Therefore, the government must have the political willpower to approve and implement a clear and thorough framework that facilitates and guides the realisation of strategies.

The above discussion shows that Uganda’s progress towards achieving UHC still needs to be increased, as many of the critical tools for achieving UHC are lagging. The WHO notes that there is a need for concrete and targeted action otherwise not many countries are likely to achieve UHC— due to the persistence of challenges such as financial and resource constraints [57], lack of political will and technological innovation, especially for developing countries [58]. However, digital health technologies are giving countries a renewed opportunity to get it right [59, 60]. Thus, digital health technologies backed with the right regulatory approach can mitigate some of the challenges highlighted and help deliver UHC, as I proceed to show.

### Universal Health Coverage and Artificial Intelligence

Digital technologies play a key role in healthcare by enhancing diagnostics, supporting decision-making processes, enabling big data analytics, and streamlining administrative tasks [16]. The arrival and deployment of AI in healthcare has been seen as a ray of light to achieve UHC as it could solve some challenges that clog the healthcare system, including the lack of health services in rural areas [61]. Technology can transform the health prospects of millions by extending the reach of services to rural communities, extending the reach of health workers, and enhancing the quality of health services. In Uganda, for example, digital X-rays equipped with AI are being used to screen and diagnose tuberculosis (TB) in key hard-to-reach areas that may not have existing health facilities [62].TB is one of the leading causes of death in Uganda and kills more than 15,000 people annually [63]. Unfortunately, people stay in the community spreading the disease and seek treatment late when the disease has already advanced. This is often due to access to health facilities and services discussed earlier. With AI X-rays, diagnosis does not need the same level of highly trained staff. Dr. Deus Lukoye, a TB expert with the US Centers for Disease Control and Prevention Uganda, reports that normally one requires an expert or radiologist to interpret an X-ray image. However, with AI, the programme interprets the image. Similarly, according to Dr. Aldo Burua, the tools are quick and swiftly interpret images enabling the screening of more than 150 people in a day [62]. This has enabled early detection and treatment of the disease.

This shows that AI is tackling some challenges that have frozen the health sector for decades, such as access to health services due to geographical differences in the availability of medical expertise. Therefore, one of the solutions to the current disparities in the healthcare workforce is to promote the use of technologies like AI [64]. In recognition of this, the government of Uganda through the Ministry of Health has invested in 22 AI X-ray systems in five mobile vans. Such initiatives will improve the diagnosis of diseases and delivery of healthcare services. Although this is a step in the right direction more still needs to be done to ensure such services reach everywhere. Uganda has 135 districts so the five mobile vans cannot cover all regions. This means that current initiatives must be enhanced through effective coordination and collaboration among national agencies and organisations. The Ministry of Health should for example establish a comprehensive strategy for integrating AI in the healthcare sector. This would not only facilitate a unified approach and enhance coherence across the field, but it would also assist in recognising and tackling existing gaps in ongoing efforts, thereby fostering significant advancements in critical issues such as infrastructure, skill development, and dissemination.

Technology also contributes to improving the quality of care. AI-powered diagnostics and health screening positively impact health service delivery [61]. Medical personnel have opined that AI can improve insights in disease modelling, diagnosis, classification and prediction consequently increasing the quality of UHC, response to emergencies and supporting healthier populations [65].The Ocular Project in Uganda is one such example [66]. The project uses AI-automated mobile microscopy to enhance the diagnosis of cervical cancer, tuberculosis and malaria in Uganda. With the help of AI, screening and diagnosis of these diseases will be more accurate, easier and quicker [67].

Additionally, AI is already helping to increase accessibility, for example, through virtual assistants that provide basic healthcare advice and triage services. For example, Dr. Shamim Nabuuma, a Ugandan medical doctor and entrepreneur has invented KETI, an AI Medical Records Interpreter that aims to ensure that medical information is accessible and understandable for everyone [68]. The AI Chatbot interprets medical records like laboratory tests, prescription notes, discharge forms and medical jargon among others. It enables users to write text or upload medical documents like laboratory tests, and prescription notes and get clear easy to understand explanations. This enables patients to understand medical terminology and the care they are receiving. With limited access to doctors especially in remote areas, this seems like a viable solution to disparities in health workforce distribution and can help fill the access gaps. Thus, AI not only has the potential to extend the reach of health workers but can also facilitate enhanced availability of information. This is useful for health planning, monitoring, and evaluation of public health programs [68]. However, this AI tool is only available via web and WhatsApp thus people with no access to the internet or smart mobile devices are not catered for, potentially contributing to existing inequalities. In addition, there is currently no specific law regulating the provision of such remote health services in Uganda. The National Drug and Policy Act was designed to regulate physical interaction between healthcare providers and patients. Thus, while such remote health services improve access, they have the potential to expose patients to harm if not well-regulated.

Therefore, while AI is a powerful tool for achieving UHC, certain challenges remain, some of which cannot even be solved by AI, such as the lack of a proper regulatory framework and the lack of political will. The use of AI in healthcare also carries challenges, for example, data privacy concerns, bias, lack of necessary infrastructure needed to run and maintain the expensive AI systems and potential risks associated with AI in health. These risks could further increase existing inequalities and instead hinder the achievement of UHC. Such challenges may further limit equity and access and exacerbate existing inequalities. As can be evidenced in the examples, many initiatives are centred in select areas and have not spread out to the whole country. The government of Uganda will thus need to develop a regulatory framework that can drive the provision of the infrastructure needed to spread initiatives across the country for example through incentivising private sector investment, increasing funding to the healthsector and having accurate data. Uganda can’t develop and execute efficient frameworks unless it has the relevant, relaible and updated data for its populace. Although a population census has recently been concluded, reports show that enumerators did not reach some areas which questions the authenticity of the data collected. Suffice it to note that formulation and implementation of policies and programs greatly relies on the availability of sufficient data. The WHO highlights that having accurate data facilitates the monitoring of health inequalities and tracking disadvantaged populations. Without this data, there is no evidence base to ‘formulate more equity-oriented policies, programmes and practices towards the progressive realization of UHC’ [5]. Therefore, Uganda needs to develop data collection tools and systems to gather health disaggregated data with more accuracy as it is essential to plan, establish and implement Uganda’s strategies for achieving UHC. However, such framework should also be balanced alongside safety and ensures that the rights of citizens are protected.

Hence, for the Sustainable Development Goals (SDGs) to deliver on their central pledge of leaving no one behind, AI will be pivotal in the achievement of UHC as has been discussed above. However, to tackle the challenges highlighted and increase AI’s benefits on health, a robust regulatory framework that considers the context of each jurisdiction in which AI is to be deployed is needed. Such a framework will help mitigate the risks and uphold the benefits of AI in healthcare if UHC is to be achieved by 2030.

States have been cautioned that realizing UHC is ‘a long dynamic, continuous, and relentless process’ that necessitates a change in approaches and flexibility adapted to shifting demographic, epidemiological, and technological trends [5]. Indeed, there have been considerable changes since the MDGs were launched in the 2000s, and a comparative analysis of the MDGs and SDGs texts illustrates this point. Whereas technology was barely alluded to in the MDGs’ text, all 17 SDGs have a digital component. This is evidence of the changing times. States need to keep up with the changing times by adopting transformative approaches, even in regulation.

Having established the benefits that AI offers for achieving UHC, we are now able to examine approaches for the regulation of AI in healthcare.

### Exploring regulatory approaches for AI in Uganda’s healthcare

Regulation has always played a significant role in monitoring and improving healthcare safety. It ensures safety and reliability in patient treatment and a safe working environment for healthcare professionals [69]. However, since its arrival, the regulation of AI has remained a key debate, with some authors arguing that regulation does not appear to be keeping up with the technology’s growth, use, and adoption [70]. Balancing innovation with control and the slow pace at which regulations are developed and passed have been highlighted as some of the inhibitors to keeping in stride with the fast-growing technology [71]. Nonetheless effective regulation plays a central role in the successful and responsible deployment of technologies including AI. Regulators around the world are thus exploring new approaches to regulating AI in various sectors of the economy including healthcare [72].This is happening amidst the debate on whether the traditional regulatory approaches and frameworks are suitable.

Hence the relationship between law and technology must not be underestimated. Technological innovation can be driven by policymaking and the policy cycle can also be driven by technology as once technology is deployed further regulation can occur [73].

Governments can use regulation as a tool to specify actions in their territories. Through it, they can promote and discourage certain activities thus protecting their citizens from harm. Regulation need not be restrictive, expensive, or burdensome [74]. It can simply mean the passing of a binding set of rules that serve as the state’s influence in the social, political and economic spheres [75]. Such reasons do warrant the regulation of AI. However as mentioned earlier, a key question remains on what regulatory approach to take.

Although AI has many positive aspects, it is not faultless. Bias and the unexplainable nature of AI systems are often stumbling blocks to public trust. Since AI systems function based on the data they are trained on, data which humans select, potential biases will often inevitably exist since humans possess their own biases [76]. This can have horrifying impacts on society if left unchecked. A case in point is the racial bias that was reported in a prediction algorithm in the US Health Care Program. Relying on earlier training data captured from an individual’s healthcare expenditure, the algorithm predicted that African American patients were more likely to be sick and need treatment compared to their white counterparts, leading to their exclusion from the healthcare program [77]. Although the bias was detected and corrected, the unpleasant incident highlights the fact that these tools reflect existing inequalities and can be discriminatory, which justifies a re-evaluation to ensure ethical use that benefits the society as a whole [78].

In addition, the unexplainable nature of some AI algorithms means that sometimes even the data engineers may not be able to retrace and understand how an AI system comes up with a particular result, a situation often referred to as a black box [79]. Governments are expected to be accountable to their citizens for the decisions they make, therefore governments cannot sit back and expose their citizens to harm or black-box scenarios. This will result in a breach of obligations and a lack of trust.

Thus, although the potential benefits of AI in healthcare are enticing, it is crucial to recognize its limitations and the associated risks that may arise during its advancement. The reliance on extensive datasets for training AI models inevitably raises issues regarding data privacy and security. Instances of unauthorized access or data breaches could significantly undermine public confidence in the innovative applications of this transformative technology. The effectiveness of AI models is intrinsically linked to the quality of the datasets used for their training [80]. As highlighted above, any biases present in the data can lead to discriminatory outcomes and further entrench existing healthcare disparities. Additionally, the complexity of algorithms may hinder interpretability, resulting in a lack of transparency in the decision-making process. This lack of clarity can prompt concerns regarding accountability and introduce new ethical dilemmas [80].

The need to create public trust in AI systems prompted the European Union and the UK to develop a regulatory framework for AI [80]. But can these approaches taken by the EU or UK deliver UHC for Uganda if applied, or will Uganda need to consider a different approach to AI regulation? To answer this, it is important to understand the concept of choosing an appropriate regulatory approach.

The decision whether or not to regulate and how it should be done is a political question, requiring a country to opt for a mechanism that best serves its interests. This will often differ from country to country or region due to differences in needs, priorities and levels of development.

A country or region has the freedom to select a suitable approach based on its specific needs and priorities, which often results in the existence of various regulatory approaches. In the AI sector, it can be argued that a developing field like AI would benefit more from an approach that provides flexibility for developers and regulators to grow, explore and understand as the technology develops. This can best be facilitated by a principles-based approach to regulation. This pro-innovation approach has been taken by the UK, as I discuss later in the paper. On the other hand, another region may choose to prioritise the mitigation of risk and harm and thus opt for a risk-based approach. However, cooperation and agreement on key terms are usually encouraged even where different regulatory approaches have been undertaken. For example, for AI, cooperation can be in the form of maintaining a register for AI risks.

Another key issue is the regulatory choice of whether to go for a ‘hard law’ or a ‘soft law’ approach. This is usually influenced by many factors, such as the regulatory objective, regulatory priorities, obligations of the regulated, and the enforcement method [81]. Since a regulatory choice will have implications on the behaviour of the regulated, the allocation of risk and whether the regulatory objective is achieved, it is important to choose an approach that will not only achieve the set regulatory objective but also attract positive behaviour (in the form of compliance) from the people being regulated. This promotes effective regulation.

Currently, the regulation of AI has mainly taken on a soft law approach as evidenced by the global pursuit to develop ethical principles over the past years. The ethical guidelines help build consensus and check whether we are on the right track toward the society we want to live in as this technology develops. The newly passed European Union “Regulation laying down harmonization rules on Artificial Intelligence” (EU AI Act) is the first attempt at a hard law approach to the regulation of AI.

The regulation of AI in the European Union has taken a risk-based approach as evidenced by the EU AI Act while the United Kingdom is leaning towards a principles-based approach as highlighted in the White Paper on AI regulation. I thus examine these two approaches below to explore whether they can deliver UHC if applied in the Ugandan context.

#### Risk-based regulation of AI: Can the EU Artificial Intelligence Act be a standard for AI regulation in Uganda’s healthcare?

Risk-based regulation is a key component of the regulatory framework in the public and private sectors. It emerged in the 1980s and 1990s following a regulatory crisis that saw the regulatory space filled with inflexible regulation that had resulted in ineffective, inefficient and costly practices in the regulatory state [82]. Consequently, it became a norm for all regulators in the United Kingdom to take into account risk as part of their regulatory regime. This began with the Better Regulation call in the late 1990s, followed by the Hampton review of 2005 and culminated into the Regulators Compliance Code [83].

Before implementing an instrument or legal framework to address an activity, a risk-based approach to regulation first identifies the risk. It selects the appropriate indicators, gathers sufficient information relating to those indicators, and assesses probabilities, management systems as well as risk mitigation processes. It also deals with other uncertainties besides risk that may arise [84]. Risk may be defined as exposure to a situation involving the possibility of injury or loss [85]. Risk is different from harm (which is present). Risk deals with future events and requires preventive measures to avoid or mitigate them [85]. In taking preventive measures, one visualizes future uncertainties, like the kind of harm that might occur, to whom it might occur, and the degree and likelihood of the harm. They range from minimal to disastrous [85].

The approach places risk at the centre of the regulatory model which compels society to explicitly anticipate risks and consider issues that might have been poorly managed or overlooked. The risk-based approach is often looked at by regulators seeking to limit the boundaries of responsibility and accountability [86]. The EU has adopted this approach to regulate AI.

The European Commission proposed the regulatory framework for AI in April 2021 [87]. This was the pioneer effort to enact a horizontal regulation for AI within the EU. The proposed legal framework (referred to as the AI Act herein) centres mainly on the specific use of AI systems and related risks in general, without being bound to a specific sector [88]. It is intended that the legislation will be future-proof and flexible, allowing it to adapt and evolve as new regulatory challenges and concerns emerge [88]. The AI Act was passed in May 2024 [89] and adopts a risk-based approach [90].

Some scholars believe the risk-based approach adopted by the AI Act is suitable for a volatile sector like AI and has often been used in regulation [91]. The AI Act introduces different requirements according to the perceived level of risk imposed by the AI system. As the risk imposed by the AI increases, stricter obligations and regulations are imposed. Hence, a lighter legal regime is levied on AI applications with limited or negligible risk while strict rules and compliance obligations are applied to high-risk AI systems [92].

The AI Act establishes four categories of risk posed by AI systems, based on a pyramid of criticality [92]. The first category under article 5 of the Act, deals with unacceptable risk. AI systems that deploy concealed techniques to distort or manipulate human behaviour are prohibited. The article prohibits AI systems that deploy harmful manipulative subliminal techniques beyond a person’s consciousness to distort a person’s behaviour in a manner likely to cause physical or psychological harm, those that exploit vulnerable groups, those used by public authorities or on their behalf for social scoring purposes and real-time remote biometric identification systems in publicly accessible spaces for law enforcement purposes, except in limited cases. The wording of Article 5 shows that the commission’s stand towards AI practices that exploit and distort humans is non-negotiable. The Act provides that such systems should neither be put in service nor placed on the market. This article protects individuals from being exposed to practices from which no legitimate benefit exists.

Article 6 of the AI Act deals with high-risk systems, the second category. These are permitted however they are expected to comply with the rigorous mandatory requirements. High-risk systems are separated into two categories: AI systems that are products or form part of a product as a safety component that is already subjected to other legislative frameworks. These products must undergo third-party conformity assessment before being placed on the market or put in service. Medical devices fall under this category. The requirement for assessment applies whether the product is sold independently or not. This means that developers of products with AI safety components will not purport to sell them separately in a bid to evade the obligations required of high-risk AI systems as the third-party conformity assessment has to be done in all respects. This is a step in the right direction. Healthcare AI, particularly in the context of medical devices, necessitates rigorous evaluations to prevent significant harm that may arise from their use if they are not properly assessed.

The second category of high-risk systems concerns those listed in Annex III. The AI Act highlights eight areas in which these systems may exist. These include AI systems in biometrics, education and vocational training, employment, law enforcement, migration, access to essential public and private services, critical infrastructure, and administration of justice and democratic processes [93].These areas are indeed the foundations of society; thus, it is justified to deem them high-risk, as unrestricted use of AI can lead to devastating outcomes and breaches of fundamental human rights. However, an exemption clause was included in this provision to cover AI systems that do not significantly risk health, safety, or fundamental rights [94]. With this exemption clause in place, certain AI systems will not be subjected to the obligations arising from the law [95].

Although information technology professionals have welcomed the exemption clause, it may be difficult to draw the line on which systems qualify for the exemption clause to be applied to them. High-risk systems are classified as such because they potentially adversely affect fundamental human rights, public safety, and livelihood if left unchecked. Thus, if a system is not likely to lead to significant harm why would it have been classed as high risk in the first place? It also needs to be clarified what the degree of likelihood to cause significant harm is which leads to legal ambiguity.

The EU Council states that the rationale for the exemption clause was to check bureaucracy and reduce unnecessary burdens [87].However, it may not be possible to determine from the onset if an AI system is not likely to cause harm. This can only be achieved if a risk assessment is undertaken, but the Act is silent on this. Much as the possibility of amending Annex III in the future exists, this will happen later after the system has proved not to pose a threat [96]. The exemption clause could thus lead to unnecessary misunderstandings especially since the Act stirs clear of defining high risk AI systems.

Another notable weakness of the AI Act is that it does not permit the introduction of new high-risk areas [96]. As noted earlier, risk management necessitates assessing the risk and then taking preventive measures to eliminate the risk. However, if the Act does not permit introducing high risk systems that do not fall within the existing criteria, this raises the question of how future threats will be dealt with. What happens in a scenario when a perceived low risk becomes a high risk due to a change in the deployment context? Will new legislation be needed to solve this issue? Or should we look at established principles to fill the gap? It may be necessary to allow for the introduction of new high risk sectors especially to protect the rights of the vulnerable or the reliance on principles to fill the gap will be inevitable. Uganda will mainly depend on imported AI systems, therefore what may be considered as a low risk in the country of development may be a high risk in Uganda due to differences in the levels of development, training and available resources. For example, an AI system has been classified as safe and secure as long as it has a functioning internet connection and a continuous power source. Suppose such a system were deployed in a rural Ugandan setting with poor internet connection and constant load shedding that disrupts power supply. In that case, chances of this becoming a high risk situation arise which could compromise fundamental human rights like the right to life. Additionally, such AI tool could be considered high risk if it exacerbates existing inequalities because it can be used to improve healthcare in better-off areas but can’t be used in more rural areas with power disruptions.

The third category of AI systems considered by the AI Act is low-risk AI systems, which are expected to be transparent in line with Article 52. All the other AI systems that do not fall under the above categories are classed as having minimal risk. They are permitted to be developed and implemented following existing legislation and have no additional obligation [88]. However, as raised above, due to differences in levels of development, these classifications may need to change in the Ugandan context. Regulators should, therefore, give due regard to the fact that risks can differ according to context. Current risks envisaged today may not adequately reflect the threats that will exist tomorrow. Some companies using AI have reported facing breaches like AI data poisoning that they had not foreseen [97]. This calls for more flexible alternatives that can address society’s needs should such uncertainties occur.

Several measures exist in the Act as requirements and obligations to promote public trust in AI systems [98]. These relate to providers, users, and other parties [99], and require quality management, documentation, conformity assessments, compliance, provision of information, and cooperation. These obligations are an onerous burden, especially on the providers and could instead end up stifling innovation or pushing developers from the market. It has been noted that innovation requires room for experimentation and risk. With the Act placing a lot of focus on ‘extensive compliance lists’ and ‘controlled innovation’ via regulatory sandboxes, the ability to innovate will be stifled [100]. In being pushed out of the market, such developers may turn to countries where fewer restrictions exist or apply. There is also the likelihood that the numerous restrictions may create monopolies as providers shy away from investing time and resources in the heavily regulated sector. The creation of monopolies will consequently affect consumers as prices become high. This is disadvantageous to people in resource-limited settings like Uganda because if we are looking to use AI to achieve UHC where one of the key components is the reduction of financial burden in access to healthcare then increased prices in AI systems will mean we can’t leverage their benefits to achieve UHC. As I observed earlier in the paper, it is the people in the rural and hard-to-reach areas who UHC stands to benefit most, thus getting AI benefits to them at the lowest possible opportunity should be key.

Some scholars have criticized the approach taken by the AI Act as being too vague, idealistic [101], and inherently deficient [102]. The Act ignores a full range of risks as it only distinguishes between high-risk and non-high-risk AI systems [102].This will be problematic as some risks may not be currently foreseeable [102]. Certain high-risk AI systems may therefore be overlooked or mistakenly identified as low-risk [102]. This may happen in a bid for developers to push their products onto the market and may be easier done in developing countries where the regulatory framework is not as strong. The regulatory landscape for technologies in many African countries is still underdeveloped. For example, there is no sui generis regulatory framework for AI in Africa and many African countries are yet to develop data protection laws [29]. Regulatory frameworks serve as a mechanism for evaluating the maturity of artificial intelligence in the healthcare domain. Consequently, the lack of well-defined regulatory guidelines and policies for AI may, in some cases, hinder the adoption of AI technologies within the healthcare industry [29].

It is also worth noting that the Act is silent on who decides the level of risks [103]. This may further exacerbate AI developers’ exploitation of developing countries due to lower bargaining power and greater need. Developing countries will mainly depend on imported AI tools due to lack of technical expertise and financial means to develop their own. This gives developers an upper hand in determining terms and conditions at the detriment of the developing countries. Invasive AI applications are being tried in Africa, capitalizing on the continent’s need for cash and the relatively lax regulations surrounding data privacy. For example, Kenya serves as the headquarters for Facebook’s ethical AI content moderation operations in sub-Saharan Africa. Employees in this role receive compensation as low as $1.50 per hour for continuously viewing highly distressing material [104]. Recent reports have underscored the detrimental effects experienced by data workers and content moderators in East Africa and South Asia due to their exposure to graphic material [104]. There are apprehensions that this exploitation may persist or even escalate. The absence of strong data protection and artificial intelligence policies in many developing countries raises the possibility of increased misuse as the influence of AI expands.

Article 10 (2)(f) of the Act, for example, highlights the need to take into account the potential biases that may exist in high-risk AI systems, it is not clear however how potential biases can be envisaged beforehand [105]. As Kop points out, thorough assessment will be essential throughout the lifecycle of AI systems because if left unchecked, these systems will be determinantal to society [92].But do resource-limited settings have the financial capability or human resources to undertake these checks? The regulatory space of many developing countries is often filled with legislative interventions based on global templates which sometimes ignore the recipient jurisdiction’s unique needs [29]. This is occasioned by a lack of resources, which causes such countries to be unable to set their regulatory agenda and end up copying and pasting from global templates.

Some AI systems used in healthcare are classified as high-risk systems such as the AI-assisted medical image diagnosis. (I discussed earlier how Uganda is using AI-powered X-ray machines to test for TB). In such a sector any errors or slips may be fatal and hazardous to human welfare and life. This means that in addition to clarity and the harmonisation of standards being key to the successful regulation of high-risk AI systems [88], so should the context in which these systems are deployed which will in most cases differ from where they have been developed. (The AI-powered X-ray machines were manufactured by Delft Imaging Company in the Netherlands). Therefore, the AI Act’s failure to clarify risk, particularly high-risk systems, should not be overlooked. Without clear guidance and classifications on what amounts to a high-risk system, developers may be unable to undertake their regulatory obligations, more so if the system has been deployed outside the country of development. While Annex III, which contains high-risk AI systems, can be amended as permitted by Article 7 of the Act, there is a need for further flexibility as these categories may apply differently in practice. This would cater for risks currently not envisioned [88].

Additionally, before using a high-risk AI system, developers are expected to undertake some procedural box-ticking to show that the AI system is compliant. However, cases of harmful systems being compliant theoretically but not practically may arise in this process of simply ticking off compliance [105]. This box-ticking mentality can create a ‘rule following mentality’ in the regulated and could make developers pay more attention to adherence to these rules as opposed to care for principles such as safety [106]. As a result, an AI system could pass all procedural aspects in the box yet remain unfit for purpose when placed on the market, especially if the market is in a different context from where it was developed.

The above analysis shows that the AI Act is a landmark step in the technological field and will influence future legislative frameworks. However, it has certain significant shortcomings and is therefore not comprehensive enough to take on as a stand-alone model to deliver UHC which is the greatest need for AI in Uganda’s healthcare. A risk-based approach may be more useful in a technical environment, not healthcare. Healthcare AI systems frequently handle sensitive personal information and are involved in crucial decision-making processes. Relying solely on a risk-based approach may not adequately ensure patient safety, particularly when accounting for possible biases and inaccuracies within AI algorithms. Additionally, AI in healthcare raises complex ethical questions that a risk-based approach may not adequately address. The future of UHC and healthcare cannot be left at the mercy of these gaps, as such gaps will expose vulnerable groups to further disadvantage and marginalization hence the need to think of another regulatory approach that will achieve the objective of promoting UHC. A successful regulatory framework for AI in healthcare will probably necessitate a multifaceted approach. This approach should not only attract AI developers to come invest in Uganda but also ensure that Ugandans are protected and able to enjoy the full benefits that AI offers in health. Thus, I consider the option of principles-based regulation as another way to regulate AI.

#### Principles-based regulation of AI: The United Kingdom position and lessons for Uganda

Principles-based regulation focuses on the use of principles as opposed to rules. The debate on whether to use principles or rules continues to date. A lot has been written about the strengths and weaknesses of each approach, especially following the global financial crisis in the UK [107–109]. The Financial Services Authority had emerged as a prominent advocate for Principles-Based Regulation (PBR) and was recognized globally as a pioneering entity in the development of PBR within the financial services sector. Hence the crisis was seen as an indication of the failure of principles-based regulation [110]. Further engagement in this debate is outside the scope of this paper. As Julia Black notes,

…the discussion to use rules, principles or standards is a policy issue in its own right.

In favour of a principles-based approach, she continues to note that,

‘…although policymakers still choose rules based…principles based evokes an image of being outcome-oriented, flexible and harbouring ethical standards [111].

The main theory behind a principle-based approach to regulation is that legislators frame high-level principles instead of imposing a set of definite behaviours [112]. It relies on these broadly stated principles to influence the industry’s development. Principles-based regulation establishes a standard to be achieved and gives the regulated more freedom in deciding how to achieve it. This is usually more acceptable in the circumstances and improves regulatory efficiency [74]. By giving the regulated entities the leeway to determine how they act to meet the regulatory objective, the regulated are more open to securing outputs [74].

Principles-based regulation can take the form of structure or content [112]. With proper implementation, it can enable dialogue and engagement between the regulator and the regulatee, which interaction creates positive outcomes such as open communication, knowledge sharing and understanding between parties. This ultimately leads to purposive and responsive regulation. A notable instance of principles-based regulation is the emphasis placed by the UK’s Financial Conduct Authority (FCA) on the principle of treating customers fairly (TCF). This approach, grounded in principles rather than prescriptive rules, has been in effect for several years and has led to substantial enhancements in how the financial services sector engages with consumers.

The best benefit of this approach to regulation lies in its flexibility. Considering the fast pace at which technology advances vis a vis the process it takes to create legislation, the approach’s flexibility makes it a great option to adopt. For example, AI is developing at faster pace than regulation and concerns exist that regulation is lagging. Although the UK believes that regulation of AI will be needed at a later stage, currently it has adopted a principles-based approach for the regulation of AI [113].

In 2021, the UK concretized efforts to regulate AI by developing a National AI Strategy, a ten-year plan for AI in the UK to make the country a ‘global superpower’ in AI [114]. The strategy also seeks to enable the country to develop the world’s ‘most trusted and pro-innovation governance framework’ [114]. Following this, in March 2023 the government published the AI Regulation White Paper [115]. The White Paper sets out the regulatory framework for AI and reiterates the country’s pro-innovation stand on AI. It outlines five principles that regulators within their specific sectors are to interpret and apply [115]. The principles are safety, security and robustness; appropriate transparency and explainability; fairness; accountability and, governance; and contestability and redress [115]. The approach taken by the UK has been applauded as being context-specific and flexible [116]. As noted earlier, this flexibility is advantageous because it allows for the interpretation of the principles to evolve as the technology evolves. This makes it more forward-thinking than the AI Act. The context-specific approach prevents the homogenous application of rules to AI systems in different sectors as benefits, risks, and deployment will also differ. As Roberts et al. note, “it is reasonable to expect a high degree of scrutiny over the use of AI systems for high-risk medical decisions, while less scrutiny may be required for other lower-impact areas, such as supply chain logistics.” [116]. Such an agile regulatory approach is in line with Uganda’s aspirations. Uganda’s Fourth Industrial Revolution strategy highlights the need for regulators in Uganda to adopt agile responses to regulation [117]. This connotes flexibility as seen in the UK approach. Such an approach may be helpful to Uganda as she seeks to leverage AI’s benefits to achieve UHC. With the regulated having the capacity to determine how they adhere to the set principles, companies working in AI will be more open to coming to Uganda to deploy their AI tools.

The White paper provides that regulators will use existing laws and regulations to implement the principles. Where necessary, targeted and supplementary regulation will be enacted in the future [115]. It is a bonus that these principles are not entirely novel; however, their particular implementation in the context of AI is a more recent development. Some of the principles have been derived from established regulatory frameworks and ethical standards across different domains. Although resource-limited settings may not have to go through the rigorous and expensive exercise of enacting new legislation to cover the deployment of AI, there is a need to inquire whether these principles are sufficient for the task. In addition, the delegation of responsibility to existing regulators who can focus on their specific sectors will lead to appropriate guidance and governance [116]. For example in healthcare, healthcare regulators are better placed to understand and give guidance on the needs of the sector and how AI can fill gaps while ensuring respect for human rights. It is undeniable that introducing legislation to an area that is novel and still developing can be challenging as balancing the need to protect people from harm while being pro-innovation is a challenging task, especially for regulators. Therefore, reliance on existing principles and existing expertise reduces this burden. Principles-based regulation and specifically reliance on legal principles is often the solution to the balancing challenge in AI regulation [118].

It should be noted however that this does not ultimately solve the problem as regulation of technology by its very nature suffers from the pacing problem (where the development and changes in new technologies outpace the ability of the law and regulation to keep up). This means that the initial response taken by regulators towards a new technology may not be sufficient to meet the regulatory objective creating legal uncertainty. This is a risky gamble to undertake in a sector as delicate as healthcare therefore Uganda will need to rethink its approach to ensure that the law is always certain and that people are protected. As has been pointed out in the UK White Paper, the need for new legislation will arise in the future due to the changing nature of technology [116]. However, this may take work for developing countries where challenges such as financial constraints and lack of political will cause severe delays in passing legislation. Uganda has bills that have been pending passing into law for over five years. For example, the Food and Nutrition Bill and the National Health Insurance Scheme Bill could greatly advance health rights, but have never been passed.

It is noteworthy that explainability is among the principles highlighted in the White paper. An AI model can be described through AI explainability, and potential biases and expected impact are highlighted. This helps in characterization of the model’s accuracy, thus promoting transparency and fairness in AI decision-making [119]. However, in black box situations (where the AI developers are unable to explain or justify the outcome of an AI decision), explainability has been held to be a likely hindrance to using AI in places with strict regulatory compliance requirements [120]. This rigid requirement for explainability has been criticized as failing to appreciate the difference in the development, deployment and governance of AI systems [121]. This translates to different AI life cycle stakeholders needing different explanations from AI models. This stumbling block may be overcome since principles-based regulation has more flexibility and less restrictive requirements for adherence to rules. A principles-based approach may allow for principles to be balanced as long as they are all considered and no eminent risk is foreseen. Take an example of an AI tool that monitors post-caesarian blood pressure being deployed in a health centre in rural Uganda, a user there will have neither the technical expertise nor desire to know how the tool operates. If it can get them monitored and reduce maternal mortality rates, then all is well. Yet in a restrictive regulatory environment, such a user may be denied access to such a tool if it fails to pass the explainability test.

A notable weakness of the White Paper is that currently there is no statutory legal duty on regulators to have due regard for the principles [115]. Although it is feared that introducing statutory duty will hinder innovation, this omission means that the regulators still need to be given an enforcement mechanism. So, what happens in a situation where there is a breach? Who will be held accountable or liable? With developing countries like Uganda being on the receiving end of technology as noted earlier clear enforcement measures must exist especially where we seek to deploy AI systems in sectors as critical as healthcare.

The White Paper further establishes a ‘central risk function’ for the government where the government will retain control to ‘identify, assess, prioritize and monitor cross-cutting AI risks’ that require the government’s intervention. This means that even though regulators are mandated to inform regulation in their sectors, the government will still oversee and intervene where necessary. It suggests a number of activities to achieve this function which includes *interalia*, having a central regulatory guidance on how to implement the principles, keeping a register of AI risks across different sectors and coordinated clarification of responsibilities of the regulators.

This will be a good and welcome strategy for promoting government stewardship in healthcare AI. In addition to being a provider and financier of healthcare, the government has to play the role of stewardship and check the actions of non-state actors. Since many of the AI developers seeking to deploy their systems will largely be comprised of the private sector, and as highlighted earlier, the private sector controls most health facilities in Uganda —the need to regulate the ever-growing powerful private sector cannot be overlooked. The increasing presence of private health facilities in Uganda is leading to a monopolistic situation, resulting in inflated charges for patients and the provision of unnecessary care. This will be worsened by the deployment of AI systems in healthcare as healthcare becomes more expensive to the detriment of the aspirations of UHC. In recognition of the fact that discrimination in access to services often exists in the private spheres, the Committee of Economic, Social and Cultural Rights in General Comment No.24 (2017) provides that the state has to protect its citizens from violations of their rights by private providers in the conduct of their businesses. This can be done by subjecting them to legislative measures or strict regulations that check their actions for example where denying access to affordable and adequate services, treatments or information by private health providers exists [122].

The White Paper also commits to creating ‘multi-regulator test beds and sandboxes’ for AI to ensure that innovators receive support from regulators in bringing their products to the market and to enable an understanding of how regulation interacts with new technologies [115]. Sandboxes allow for experimentation within a controlled environment. Although the AI Act also provides for regulatory sandboxes in Article 57, this has been criticized as being limited in terms of oversight, market access and scope [123]. This is because participation does not offer guarantee of market access. Additionally, although national competent authorities have supervisory powers, their ability to intervene is limited. Jon Truby et al. believe that the limited duration and liability exposed regulatory sandbox proposed by the EC is not appropriate to balance the liability risks of AI, the cost of regulatory compliance and the need to encourage innovation. Picking a leaf from the concept of Fintech and using a more unified and robust regulatory sandbox framework for AI would have served a better purpose [123].However, the UK approach encourages more cooperation between innovators and regulators, which is an essential aspect when dealing with a new growing technology [116].

It should be noted that to achieve a balance between exploiting the benefits of AI for all Ugandans and the different private entity interests concerning the development, use and adoption of AI, a state must have a proper regulatory framework within which ‘responsible’ deployment takes place. This depends on various measures which are put in place to regulate ownership, control, and access to technology. All these measures must act in concert to ensure the existence of a proper regulatory framework; that balances the rights of all interested parties and ensures that the country benefits from deploying these technologies. Therefore, as AI advances, it is essential to establish positive cooperation with innovators through an approach on sandboxes like that taken by the UK. This will promote innovation while safeguarding accessibility and affordability for all individuals.

The following table provides a summary of benchmarks that are used to compare the two regulatory approaches.

**Table:** 

Benchmark	Risk-based approach	Principles-based approach
Feasibility	It is feasible to implement in the short term, especially for AI applications with clear, predictable risks, such as medical devices.	It is feasible due to its flexible nature; however, it faces the challenge of translating general principles into applicable standards and ensuring their relevance over time.
Adaptability	It can leverage existing regulatory frameworks and standards, making it easier to adapt to specific applications. However, defining and classifying risk across a vast and evolving landscape of AI applications can be challenging and require ongoing updates.	It offers great adaptability in the long term due to its flexible nature. This is key for the evolving nature of AI as it can ensure that regulation remains relevant as the technology progresses.
Access to health	May limit access in cases where stringent regulations delay the development or deployment of AI tools.	Can foster an equitable and accessible health system by ensuring that AI is used in a way that is beneficial to all. Can encourage a focus on AI solutions that serve the needs of the underserved populations.
Enforcement Mechanisms	It is based on mandatory compliance requirements for high-risk AI, with potential penalties for non-compliance. This approach can be more easily enforced through targeted audits and certifications. However, enforcement can be complicated when AI risks are not immediately apparent.	It requires a wider approach to enforcement, which could include regulatory oversight, industry self-regulation and public awareness. However, such collaboration and coordination are needed to drive the AI revolution.

The above analysis demonstrates that the UK’s approach to AI regulation is more desirable than that of the EU because of its flexibility and agility. As noted earlier the approach’s flexibility means that principles like safety, and security can be interpreted in the Ugandan context. Additionally, provisions like the central risk function of government will enable the government of Uganda to exercise its stewardship role as AI is deployed. However, the UK approach is also not flawless as highlighted in the weaknesses. In light of this, I believe that Uganda’s healthcare sector will benefit more from AI if it adopts a mixture of a principles-based and a human rights-based approach to its regulation. This is key in ensuring that the regulatory practice and human rights principles are at par. Human rights principles such as non-discrimination, equality and fairness will be key in ensuring that AI benefits all. This hybrid approach can ensure that context-specific approaches are taken. It is pertinent to consider the specific needs of Uganda’s healthcare sector if UHC is to be achieved. As noted earlier, focusing on ensuring access, availability, protection from tech harms and ultimately upholding human rights is necessary. Considering this, I make a case for a human rights-based approach to be adopted as well.

#### Towards achieving UHC in Uganda: The case for a human rights-based approach to AI regulation

As discussed earlier, there are different modalities of regulation. However, the central goal of regulating AI in Uganda’s healthcare should be to achieve UHC in line with SDG target 3.8. This will be achieved when the four components of UHC discussed earlier (access, equity, no financial burden, and quality) are met. Consequently, rather than concentrating solely on one approach (as previously mentioned, I believe the principles-based approach is more appropriate than a risk-based approach), Uganda should also capitalize on the advantages provided by a human rights-based approach. In this way, the country can harness the strengths of both the principles-based and human rights-based approaches to maximize the benefits of artificial intelligence in healthcare and attain Universal Health Coverage (UHC).

Adopting a human rights-based approach involves placing the rights of individuals at the forefront when formulating and implementing policies and practices. Often times there has been a focus on technical compliance as opposed to the incorporation of rights based approaches. When it comes to AI in healthcare, a rights-based approach guarantees that the technology is directed towards areas where it can make the most significant impact. (having the right tools, at the right hospital at the right time). The PANEL principles of Participation, Accountability, Non-Discrimination, Empowerment, and Legality [124], offer a framework to elucidate the practical implications of the human rights-based approach. When strategizing to guarantee UHC, it is essential to take into account the PANEL principles. These principles prioritize a person-centred approach in efforts and ensure that assistance is directed towards those who require it the most. As highlighted earlier, many disparities exist in the provision of healthcare services for the people living in urban areas and those in rural areas. Hence human rights should be the foundation for UHC if the barriers to accessing health services are to be dismantled [125, 126]. If AI is to deliver UHC the primary focus of its regulation should be grounded in human rights principles like non-discrimination, participation, and accountability. Through the human rights framework, state and non-state actors have been able to develop, implement, and oversee health programs. For example, the interrelatedness of human rights, the right to health, and freedom from discrimination have been used to tackle diseases like HIV/AIDS, tuberculosis, and COVID-19, among others [127]. A similar approach should be adopted for AI in healthcare.

The right to enjoy the benefits of science and the right to health both highlight the core obligation of non-discrimination (which is an immediate obligation). Thus, any regulatory approaches should re-emphasise this and be applied to the State party obligations to advance health. Health law and medical ethics both highlight the fact that access to health care is a basic right which States have the obligation to provide for their citizens in a non-discriminatory manner [10]. The State in fulfilling its obligation to safeguard and promote the right to health, is required to implement legislative, regulatory, and other measures that guarantee the availability, accessibility, and quality of health services for all individuals. Furthermore, it must ensure that the actions of third parties do not violate the health rights of its citizens [10]. These obligations are pertinent to the discussion on AI use and deployment in Uganda’s healthcare.

In addition, States endorsed the Guiding Principles on Business and Human Rights [128] as being crucial to the achievement of the 2030 Agenda. They need to adhere to the pertinent human rights principles and standards, including non-discrimination, equality, and the right to be free from exploitation, in the delivery of health services by private healthcare providers. As previously highlighted, the private sector serves as a more significant provider and financier of healthcare in Uganda compared to the government, controlling 55% of health facilities. This makes such principles crucial in the discussion on AI in Uganda’s healthcare. An unchecked private health sector has often led to the monopolization of healthcare services resulting in the charge of exorbitant fees to the public. A case in point was during the COVID-19 pandemic when private hospitals charged up to One hundred eighty million Uganda shillings (equivalent to £35,000) for treatment leaving families in financial distress. Unfortunately, in some instances upon the death of the patient, hospitals would withhold the corpse to demand payment from the family [129]. This must be checked. As we move into leveraging the benefits that AI offers in health, we risk the private sector taking over and overcharging for services, which will defeat the whole benefit, as the poor will be left out. For Uganda, since healthcare is mostly delivered by private entities, a framework centred on human rights becomes necessary to hold them accountable and promote performance improvement. This will ensure that the intrinsic linkage between health and human rights is at the forefront of AI regulatory approaches.

As highlighted by the COVID-19 pandemic, there is a need for reform in the regulation of healthcare. Post-pandemic policymaking should focus on people-centred approaches that put human rights at the centre as opposed to disease-specific ones [130]. In Uganda, health policies are fragmented and disease-specific, which has often not achieved meaningful results. For example, despite the existence of the HIV Prevention and Control Act, which criminalizes transmission of HIV, infection rates have recently been reported to increase. A recent report highlights that as of August 2024, the HIV prevalence rate in Uganda is 5.5%, up from 5.1% in 2023, with over 700 new cases reported daily, especially among girls and young women [131]. The rising cases despite the existence of a law call for a re-examination of the approaches taken in some of our legislation.

It should also be remembered that the need to regulate AI stems from the necessity to protect human rights. It is evident that with the deployment of AI systems, fundamental rights are at stake if systems are left unchecked. Additionally, technology is always changing and thus creates new legal and regulatory problems [29]. This requires regulators to think beyond risk to other factors as well, especially since risk can change according to context. Hidvegi et al., for example, argue that the regulation of AI should be based on rights [132].The human rights framework is well established, for example, the core principle of a rights-based approach is that all people have rights by virtue of being human. It is also essential that an individual’s enjoyment of rights, as well as their access to justice for the protection of those rights, remains unaffected by their identity or origin. Therefore, the utilization and enhancement of such an existing framework, which includes defined terms and procedures and essential accountability mechanisms, is imperative. Human rights law can fill the gaps and provide a safety net for individuals. As technology changes, human rights and people’s entitlement to protection will not change. For example, a non-discrimination provision established at the beginning – emphasizing that all individuals are entitled to equal access to rights and protections will remain amidst changes in technology.

Hidvegi et al., further suggest that for proper safeguards to exist, a human rights impact assessment should be mandatory before an AI system is deployed. This assessment would aim to check that a system will not violate fundamental human rights [132].The rights-based approach to regulation is thus more adequate for protecting human rights. It makes the protection of human rights the primary agenda as opposed to other approaches whose central focus is mainly on protecting innovation [132].

A notable weakness in the UK White Paper on AI Regulation for example is its failure to explicitly mention human rights among its principles, which was also highlighted during the stakeholder consultations [115]. Human rights are essential to ensure that the benefits of AI are accessible to all. Additionally, the achievement of UHC is tied to human rights as UHC by its very nature is grounded on rights such as equality, non-discrimination and health. The 2019 United Nations General Assembly (UNGA) political declaration on UHC also recognises that the need for privacy, equity, narrowing the digital divide and promoting health priorities should be central in the use of digital technologies to achieve UHC. This will not only minimise the harms associated with digitization, but it will also promote respect for human rights, ethics and equity. This shows that human rights should form the basis of principles in AI regulation as explicit recognition of human rights is essential to achieve UHC.

Relatedly, the WHO in its different guidance [133–135]on transforming to digital technologies has continuously re-echoed the need to put rights at the forefront of the digital health agenda. It highlights strategies that should be taken to achieve this, including *interalia* protection of human rights through ensuring the existence of laws that respect human rights, justice, and equality.

A human rights approach can foster coherence and solidarity in the protection of individuals’ rights by addressing underlying structural inequalities, including poverty, discrimination, and insufficient access to essential services. This, in turn, encourages the development of sustainable and enduring solutions suited to the country’s needs. In Uganda, regulatory frameworks from other countries have been instrumental in defining and shaping the current regulatory landscape. For example, a significant portion of the legislation is derived from English law, reflecting the historical context of the colonial era. It should be noted however that “imported regulation” may sometimes be ineffective in considering and solving the unique needs of a country. In AI systems questions of imported bias, discrimination and algorithmic injustice have been highlighted [29]. Uganda should therefore ensure that she follows the regulatory objectives and that any approach chosen benefits Ugandans and prioritizes them. For AI in healthcare, ensuring equal access to AI benefits should be at the centre of the regulatory objectives. As highlighted earlier in the paper, equal access is a major obstacle in the healthcare sector for Uganda. This is something that regulation needs to address if we are to achieve the SDG targets and leave no one behind. In developing countries, challenges such as resource constraints, the digital divide, and weaker bargaining power in the purchase and use of technology make a comprehensive regulatory framework crucial as it guides the management of relationships and dependencies between governments and various stakeholders. These may include donors, health workers, the private sector, software developers and non-governmental organizations [70]. In Africa, for example, the varying disparities in infrastructure across the continent necessitate increased resources to bolster AI systems. In response, several governments have established partnerships with technology firms such as Google and IBM to promote AI development [136]. Nevertheless, while these collaborations are essential, they have sparked concerns regarding data sovereignty and the potential for neo-colonialism to resurface [136].Reports indicate that data gathered from Africa by these technology companies is often stored overseas, subject to the legal and regulatory frameworks of those regions [137]. This situation raises significant issues related to data control and sovereignty, along with apprehensions that Africans may not reap the benefits of the data collected, as it could be utilized to train AI systems that serve other interests [137]. In light of this special attention needs to be paid to human rights. This impacts my decision to advocate for implementing a hybrid approach that combines principles-based and rights-based frameworks in Uganda’s healthcare system. Such an elaborate framework is key to unlocking the benefits of AI in healthcare, especially at the grassroots level where the need is greater [138]. With human rights at the centre of policy and programming, the government can ensure that people get the best from the deployment of technology. This can be done by ensuring that a skilled and motivated health workforce exists in underserved and hard-to-reach areas [138] and that the necessary tools are available. Resources of this nature are essential to guarantee that the benefits of AI are accessible to these marginalized groups, who frequently constitute the most impoverished segments of society and are the most significantly disadvantaged. It should be remembered that the SDG aspirations call for leaving no one behind, starting with the furthest behind.

Additionally, a people-centred strategy for overseeing the benefits and effects of AI technology on individuals necessitates a human rights-based framework that emphasizes examining each person’s unique needs, experiences, and identities, which promotes context-specific approaches. In Uganda for example, the greatest need for the rural populace is equal access to healthcare. One method to guarantee and enhance equal access is by implementing bottom-up strategies in healthcare delivery. This approach will subsequently reinforce Primary Health Care (PHC). Primary Health Care is essential in achieving UHC as it ensures the delivery of well-coordinated, high-quality, accessible and comprehensive healthcare even in times of crisis [139]. In recognition of this, States adopted the Declaration of Astana in 2018 reaffirming their commitment to health for all by achieving UHC in line with the SDGs. In addition to providing health services that are safe, accessible, available and affordable for everyone, States committed to providing a conducive environment for individuals and communities to engage and be empowered to maintain and enhance their health and well-being— acknowledging that strengthening PHC was the most inclusive and efficient way to do this [140]. States acknowledged that various interventions are essential to implement PHC successfully. Many of these are bottom-up approaches such as empowering individuals and communities, capacity building, leveraging technology and providing adequate finances to the PHC sector [140]. A human rights-based approach is best suited to support this as it emphasises participation, empowerment, non-discrimination and equality.

Having made a case for a hybrid of a principles-based and human rights-based approach to AI regulation in Uganda’s healthcare, I move to propose a framework to guide the implementation of this hybrid approach.

### Proposed regulatory framework for AI in Uganda’s healthcare

The Lancet Commission recognises that laws and regulations are effective tools that can advance health and equity and, ultimately, UHC for the population as they provide both mandate and the tools needed. Unfortunately, they are poorly understood and considerably underutilized by States [30]. It is time this changed. States should use the law and regulation adequately recognising that it has the power to achieve global health with justice [30]. Thus, these ‘legal determinants of health’ should not be ignored in any discussion on health.

The 2019 Lancet Commission on Global Health and the Law coined the concept of “legal determinants of health” to recognize the law’s capacity to enhance health and safeguard rights [141]. A conducive legal framework is essential for realising UHC, especially in developing countries like Uganda. Gostin believes UHC can only be attained through the law, as it is uniquely powerful [141]. Thus to realize UHC by utilizing the law as an instrument, it is essential that all associated mechanisms, including AI, be governed by legal frameworks that prioritise safeguarding rights. This ensures that every aspect of UHC is aligned with legal standards, promoting accountability and ethical practices. Additionally, in light of the gaps regarding the future of AI, a cohesive and uniform strategy for safeguarding individuals’ rights across the health sector would be more effective. AI is a rapidly developing field with many unknowns [142]. We do not fully understand how it will impact healthcare in the long term. This uncertainty makes it difficult to predict potential risks to individual rights. This makes a unified approach to safeguarding individual rights essential to ensure that AI is used responsibly and ethically in healthcare — to protect people’s rights and ensure that they can benefit from the potential of AI without fear of harm.

#### Overview of current regulatory landscape for technology in Uganda’s healthcare

The Government of Uganda has previously identified three areas where emerging technologies can play a vital role in advancing quality health care. These include the extension of Health Information System (HIS) interoperability to create a foundation upon which further innovations can be developed [117], the enhancement of medical supply chains with technology −to overcome challenges in accessing hard-to-reach communities and improve the availability and integrity of medicines and medical supplies [117] and −improving the quality of primary health care by augmenting the capabilities of staff regardless of location [117]. To achieve the above, Uganda needs to have an enabling environment for responsible adoption of emerging technologies. The critical components of an enabling environment include a sturdy yet permissive legal and ethical framework, sufficient funding, relevant infrastructural development and improving the capacity of the health workforce.

Currently, Uganda does not have explicit laws or regulations that specifically govern artificial intelligence. This means that there are no distinct or particular responsibilities assigned to developers, users, operators, and deployers of AI systems. Nevertheless, there are compliance obligations associated with the provision of healthcare that can be utilised to ensure that the use of AI is ethical, inclusive and safe.

Uganda has recognised the indispensability of technology to its UHC aspirations by passing a National eHealth Strategy (2017–2021), a National eHealth Policy (2016) and the Health Information and Digital Health (HIDH) Strategic Plan for the years 2020/21 to 2024/25 [143]. The above initiatives acknowledge that technology is a critical enabler for achieving the long-term goals of UHC. The National eHealth policy enables the Ministry of Health to tap into the use of technology to transform healthcare delivery by providing information accessibility and support operations. In May 2023, the Ministry of Health introduced the Uganda Health Information and Digital Health Strategic Plan (HIDH) for 2020–2025. This initiative is designed to enhance the health information ecosystem within the nation by encouraging the responsible utilization of digital tools for the documentation and sharing of patient data, while ensuring effective coordination among all stakeholders. The HIDH outlines the strategic framework and key initiatives aimed at enhancing health information management and the digitization of the Health Information System (HIS). The effective utilization of ICT will enable better outcomes and create an enabling environment for the development, deployment, and utilisation of sustainable ethically sound, and harmonised initiatives at all levels.

There is, however, a major need for deliberate and strategic guidance on how to adapt to emerging technologies like AI safely, securely and gainfully. A Gap Analysis of the Policy, Legal and Regulatory framework for Uganda’s Information Communication and Technology (ICT) sector conducted in 2019 specifically identified the need to draft and adopt an infrastructure-sharing policy and a policy on Open Data [144]. These policies would directly impact the responsible adoption of emerging technologies in service delivery including healthcare, but they have never been completed.

In addition, Uganda passed the Fourth Industrial Revolution (4IR) strategy [117] to promote responsible adoption and utilization of 4IR technologies. The strategy seeks to achieve this through the recreation of an extensive framework that identifies key opportunities that Uganda can immediately harness to maximize 4IR technologies. One of the critical enablers that the strategy identifies is the need for regulatory agility. This agility requires regulators to respond to changing conditions quickly, adjust regulation accordingly and enable business experimentation with new technologies.

Further, the National Strategy recognises the need to close regulatory and legislative gaps that emerging technologies create. This is vital because the existing regulatory frameworks were designed for the Third Industrial Revolution (3IR) and may, therefore, need help tackling some of the changes brought by the 4IR [117]. Harmonizing the regulatory framework with emerging trends is needed to facilitate the safe adoption, use, or deployment of these technologies in service delivery, including healthcare [117]. This will achieve the recommendations of the Gap Analysis Study on Policy, Legal and Regulatory Environment for Uganda 2018 [117]. As noted earlier, the Gap Analysis Study highlighted the lack of strategic direction.

This section of the paper hopes to contribute to offering strategic direction in the healthcare sector by proposing the following as a regulatory framework.

**Table:** 

Proposed Regulatory Framework for AI in Uganda’s Healthcare		
1.	Objectives	The objectives of the framework will be to • Guarantee the safety, effectiveness, and ethical application of artificial intelligence in the healthcare sector. • Safeguard patient rights, confidentiality and the security of their data. • Encourage innovation alongside the responsible advancement of AI technologies. • Create explicit standards for the development, evaluation, and implementation of AI systems.
2.	Scope	The framework will apply to all AI systems used in healthcare, including but not limited to • medical image analysis software, • diagnostic tools, • treatment planning systems and • decision support systems.
3	Regulatory oversight	The Ministry of Health will provide regulatory oversight. The Ministry drafted a digital health policy which has not yet been approved. This process should be prioritised to give the mandate to the ministry. Current regulations within the health sector are overseen by the national judicial system and the professional councils, both of which may not have sufficient expertise in the realm of digital health. I propose that the ministry take the lead and work in collaboration with the professional councils.
4	Key regulatory principles	The following principles may be applied to balance innovation with patient safety, public trust and human rights. • Non-discrimination, • equity, • equality, • data privacy and security, • fairness, • accountability, • transparency and explainability, • patient safety and wellbeing,
5	Key stakeholders (who do the guidelines apply to)	The guidelines would apply to 1. Healthcare providers as implementers who use AI to deliver health services 2. AI developers
6	Key components of the framework	The following can form the basis of the components of the framework. • Key definitions • Pre-market review mechanisms to assess safety, efficiency, human rights and ethical implications • Post-market surveillance to ensure continuous monitoring of AI systems. • Human rights and ethical review boards to guide responsible AI practises. These can comprise members with expertise in AI, ethics, law, health and human rights. • Enforcement mechanism to check non-compliance. These can take the form of criminal, and civil penalties like fines and professional sanctions. • Collaboration between public and private stakeholders in the healthcare sector and international cooperation and collaboration to enable knowledge exchange

Pending the adoption of a regulatory framework for AI, Uganda can make use of the existing legal framework. Uganda has several laws and policies that can advance the right to health. It can thus leverage these to operationalise the above framework. It may not be possible to pass a National AI law currently, and indeed the process to pass one should not be rushed. However, this does not mean AI deployment cannot be done based on existing laws and policies. As observed from the UK approach to AI regulation, this is possible. Recognising the role of existing laws in AI deployment is therefore key. Regulators should seek to use the arm of the law through the existing legal principles. Below I highlight the existing laws that Uganda can make use of.

**Table:** 

Key Legislation		
	Legislation	Regulatory Context
1	The 1995 Constitution of the Republic of Uganda	Contains the Bill of Rights in which rights such as freedom from discrimination, equality, privacy, the right to life. Established the Health Service Commission which is charged with reviewing the terms and conditions of service, standing orders, training and qualifications of health workers.
2	Data Protection and Privacy Act 2019 and Data Protection and Privacy Regulations 2021	Responsibilities regarding the safeguarding, gathering, utilization, dissemination, and accessibility of personal information.
3	Electronic Signature Act 2011 Electronic Transactions Act 2011 and Electronic Transactions Regulations 2013	Responsibilities for online transactions
4	The Medical and Dental Practitioners Act,	Establishes the Medical and Dental Practitioners Council to monitor, supervise and maintain professional medical and dental education standards. This includes protection of the public against abuse by ensuring that professional ethics relating to doctor-patient obligations are maintained.
5	The Nurses and Midwives Act, Allied Health Professionals	Establish professional councils to monitor and supervise health professionals in these sectors.
6	The Pharmacy and Drugs Act	consolidates the law relating to regulation of the pharmaceutical profession. The Act establishes the Pharmaceutical Society in Uganda which is charged with enforcing standards, regulating the conduct and discipline of pharmacists and training institutions.
7	The National Medical Stores Act	established the National Medical Stores that is charged with the procurement and distribution of essential medicines and medical supplies to public health facilities
8	The National Drug Policy and Authority Act	Establishes the National Drug Authority(NDA), which authority regulates the manufacture, importation, distribution, and sale of drugs and pharmaceuticals. The NDA also exercises regulatory controls over medical devices for example licensing, product registration.

The above table shows that Uganda’s regulatory framework for health is fragmented, with multiple laws and regulations governing different aspects of the sector. This can have a number of challenges such as conflicting mandates leading to difficulty in compliance and implementation. For example, the regulatory pathway for medical devices in Uganda has been described as unclear and a burden to innovators [145].In addition to there being different regulatory bodies to which innovators must go for different permissions and approvals i.e. National Drug Authority (NDA), the Uganda National Council of Science and Technology (UNCST), Uganda National Bureau of Standards (UNBS) and the Ministry of Health, innovators reported inefficiencies and irregularities at the regulatory bodies [145]. Innovators are keen on having a clear regulatory framework, as it simplifies their entry into the market. Consequently, it is crucial to address these regulatory hurdles, as they could hinder Uganda’s potential to harness the benefits that artificial intelligence offers in the pursuit of universal health coverage.

## Conclusion

A proper regulatory framework for AI in healthcare can play a vital role in achieving Universal healthcare. I have proposed a hybrid of the principles-based and human rights-based approach to regulating the technology. This hybrid approach is best suited to serve Uganda’s healthcare needs. In the realm of healthcare, the foremost priority for Uganda is to attain Universal Health Coverage. The paper has illustrated how AI can serve as a valuable asset in this pursuit. Undoubtedly, the regulation of AI is crucial in guaranteeing equal access for all. However, this is not the silver bullet. AI and its regulation cannot work alone. They must be supplemented with different strategies through the exploration of non-regulatory approaches like increasing funding to the health sector. Uganda must also consider these strategies to achieve transformative change in its health sector and make universal health coverage a reality.

## Data Availability

No datasets were generated or analysed during the current study.
